# Association between physical demands, skin temperature and wellbeing status in elite football players

**DOI:** 10.1038/s41598-023-40396-y

**Published:** 2023-08-23

**Authors:** Carlos Majano, Jorge Garcia-Unanue, Ismael Fernández-Cuevas, Víctor Escamilla-Galindo, Antonio Alonso-Callejo, Javier Sanchez-Sanchez, Leonor Gallardo, Jose Luis Felipe

**Affiliations:** 1 IGOID Research Group, Physical Activity and Sport Sciences Department, University of Castilla-La Mancha, Avda. De Carlos III S/N, 45071 Toledo, Spain; 2 Research Department, ThermoHuman (R&D Department, ThermoHuman, Spain), Madrid, Spain; 3 Faculty of Sciences for Physical Activity and Sport (INEF), Universidad Politécnica de Madrid, 28040 Madrid, Spain; 4 School of Sport Sciences, Universidad Europea de Madrid, 28670 Villaviciosa de Odón, Spain; 5 Performance Analysis Department, UD Las Palmas, 35019 Las Palmas de Gran Canaria, Spain

**Keywords:** Anatomy, Health care, Medical research, Materials science

## Abstract

The demanding nature of elite football requires players to be closely monitored to ensure optimal performance and minimize injury risk. This study aimed to evaluate the relationship between physical demands, skin temperature, and well-being status in 30 elite football players over a 12-week competitive period. Thermography assessments, weekly Well-being questionnaires, and daily training and match load recordings were used to gather data. Results indicated that along the microcycles there was a decrease in high-intensity accelerations and decelerations distance completed, while maintaining other high-intensity actions. Furthermore, it was found that high-intensity movements contribute to the generation of thermal asymmetries in the thighs; the adductor thermal asymmetry showed a positive relationship with stress and muscle soreness, the knee thermal asymmetry had a positive relationship with fatigue and a negative relationship with rest and quality of rest, and finally the hamstrings muscles exhibited significant differences between the thermal asymmetry groups, with the high asymmetry completing less high intensity actions than the low asymmetry group. In conclusion, this study highlights the interconnections between physical demands, skin temperature, and well-being in elite football players and provides valuable insights for coaches and trainers in their efforts to optimize performance and health.

## Introduction

Football is an intermittent team sport that demands high intensity actions like tackles, shoots, jumps, accelerations, decelerations, changes of direction and dribbles, alternating with low intensity actions and resting periods^1^. The combination of those actions is highly physiologically demanding and leads to the activity of the aerobic and anaerobic systems of the players^2^. Therefore, it is crucial to understand and monitor those actions in order to improve performance and reduce the injury incidence^3^.

In the last years, load monitoring has progressed through the use of Global Positioning Systems (GPS). This technology allows the quantification of training and competition loads -known as external load- providing a great amount of data that needs to be interpreted^4^. The measurement and interpretation of these values can provide information about current physical condition of each player, guiding coaches to program training schedule in order to reduce injuries and maximize players performance^5^.

However, the values provided by GPS might lack some information about the players physical and cognitive circumstances, so other methods should be used in combination with GPS to have insights about the internal load. For this reason, the measurement of health and recovery indicators is now a common procedure in professional football^6^. For the past decade, researchers have used wellness questionnaires to monitor the condition of athletes across a variety of sports and competitive levels^7^, with questionnaires including RPE, stress, muscular pain, sleep quality and duration^8^, as sleep disturbances increase the risk, prevalence, and severity of musculoskeletal injuries^9^. Nevertheless, even though questionnaires are simple to administer, they are highly subjective and some players might modify their responses in order to play more, and the presence of a partner can alter athletes perception^10^ making questionnaires a measurement with high risk of bias whose results must be interpreted with caution^11^.

This is why infrared thermography (IRT) might be considered as an objective alternative to measure internal load. IRT is a non-radiating, contact-free, safe and non-invasive technology that monitors physiological variables through the measurement of the skin temperature^12^. Healthy subjects are supposed to keep a consistent thermal balance in neutral situations^13^ but the metabolic, biomechanical and physiological demands of the training and competition loads might cause changes in the skin temperature from the different body regions (e.g., joints, muscles, etc.). Skin temperature asymmetries in bilateral regions are associated with injury-related factors^12^. IRT can detect these asymmetries and reveal potential risks by comparing bilateral body regions^14^. The relevance of IRT is its ability to quickly identify with data those imbalances before other markers such as visual analogue scale (VAS) for pain do, enabling professionals to proactively perform training and treatment interventions in order to reduce injury risk and improve performance^15^.

Despite the small amount of literature about the application of IRT in high performance athletes, we find some studies performed with professional football teams using IRT to reduce injuries and improve performance^15–17^. Moreover, there are two studies that related IRT outcomes and sources of external information (such as GPS)^18,19^, nevertheless the sample of these investigations was very low and did not related IRT outcomes with subjective internal load such as wellness status, so evidence is very limited.

Thereby, the aim of the study is to analyse the association interaction of the thermal profile, physical demands and wellbeing in elite football players.

## Material and methods

### Participants

A total of 30 male professional football players from a professional team of the Smartbank League (Spanish second football division) (age: 25.37 ± 3.60 years; body mass index: 23.06 ± 1.04; height: 178.90 ± 5.69 cm; body fat 7.88 ± 0.91%; VO_2peak_ 61.40 ± 2.93 ml/min/kg) were included in the study. When a player had an injury, for the following weeks his data was excluded from the analysis, after recovering from injury players could be reintegrated into the analysis, it was agreed that to be reintegrated in the analysis players needed approval from medical staff of the team and train normally with the team at least 3 days. So, this way 30 players were into the analysis but not every player was analyzed every week. All subjects were informed of the protocol and purpose of the research as well as the associated risks and signed the informed consent to participate in the study.

### Ethical statement

The study was conducted according to the requirements of the Declaration of Helsinki (2013) and was approved and followed the guidelines stated by the Ethics Committee of the European University of Madrid (CIPI35/2019). The research also received formal approval from the professional football club involved.

### Design

During a 12-week competitive period throughout 21/22 season (14th February–11st May) football players underwent a thermography assessment the morning three days after the game (MD-4). In addition, training and match load was collected daily and the wellness questionnaire was fulfilled weekly by each player. Participants trained five times a week and played once a week in the domestic competition. So, a normal microcycle was compose by MD-4, MD-3, MD-2, MD-1, MD, MD+1, and then a free day. Across the data collection period, the team participated in 11 official matches. Data was gathered every week and prepared for the analysis to avoid mistakes in the final analysis.

### Data collection

#### Thermography

**Figure 1:**
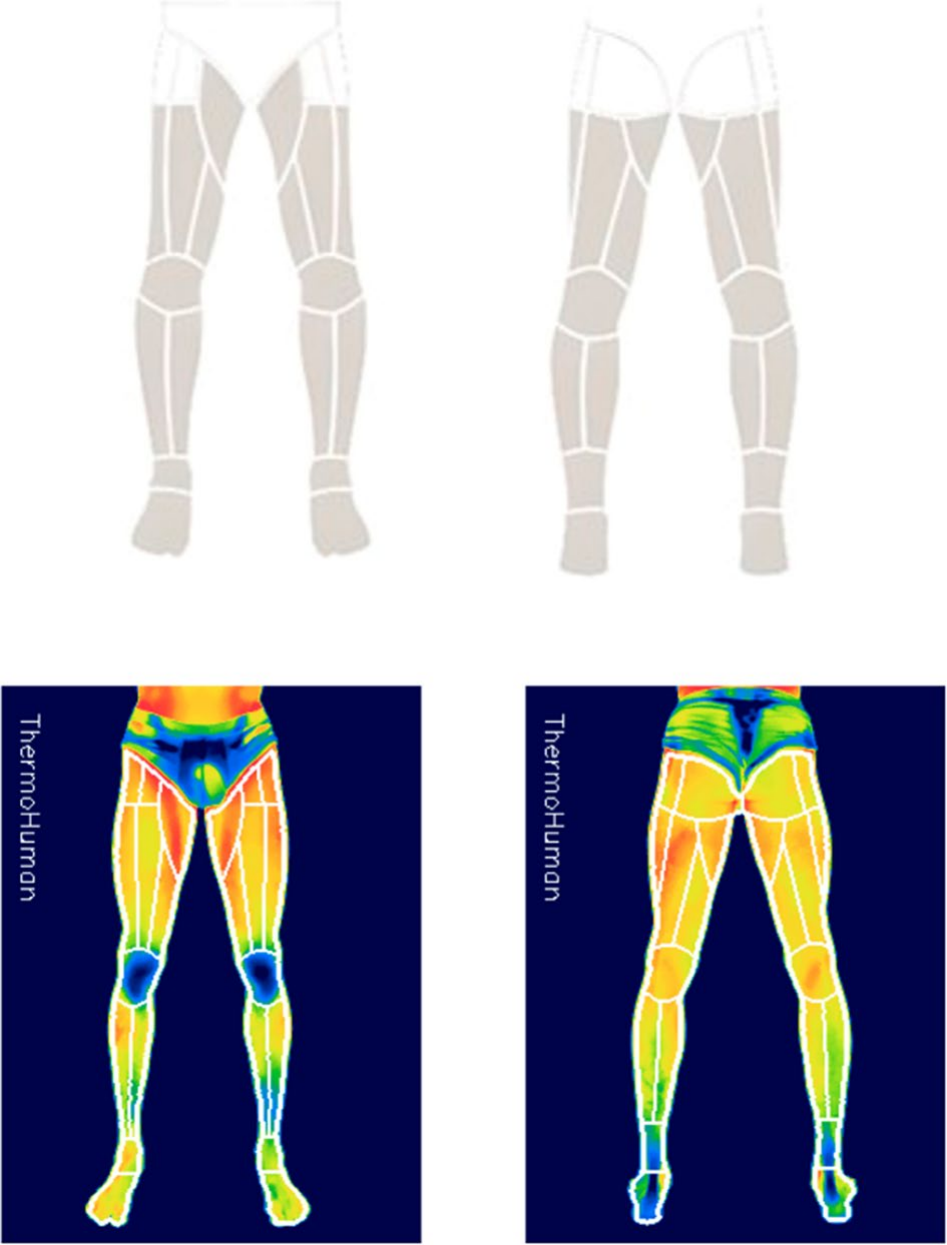
Measurement areas provided by the Thermohuman® software (anterior and posterior parts) and segmentation.

For 12 weeks all the available players of the team were analyzed by the same thermography technician under controlled conditions. Normally, athletes played the match day (MD), trained the next day (MD+1), had a resting day (MD+2), and after the resting day they had the thermography analysis session in the early hours of the morning (MD-4) as soon as they arrived at the training facilities. The thermography session day was chose based in that research findings suggest that soccer players are more likely to experience muscle injuries during games compared to training sessions, has been observed that a recovery period of approximately 48 h is required to alleviate delayed onset muscle soreness and reduce creatine kinase activity^20^. The collection of thermographic data followed the standards proposed by the consensus statement of TISEM on the measurement of human skin temperature^21^. Thermograms were performed in a controlled room, where the ambient temperature was set at 22 °C ± 1.5 °C with about 40 and 60% of relative humidity^21^. The thermal camera FLIR T435bx (FLIR Systems, Sweden) with a resolution of 320 × 240 pixels and thermal sensitivity =  < 0.04 °C/ < 40 mK, was placed 3 m away from the participants and at a perpendicular angle to them, around 60 cm height. The players were instructed to rest 24 h prior to the thermograms and to avoid behaviours that could interfere with the assessment of thermal images, like drinking alcohol, smoking, consuming caffeine or manipulating the skin (e.g. with ice, massage, ointments, etc.). During testing, the participants were dressed in underwear and were barefoot, so selected areas of skin were continuously exposed during the measurements. Two thermal images were taken following the Glamorgan protocol^22^ for each player, one for the anterior part of the lower limb and another for the posterior part of the lower limb, recorded by the thermography technician. Thermal images from the anterior and posterior lower limbs from the players were analyzed with ThermoHuman software version 2.21 (Pema Thermo Group, Madrid, Spain), which is a validated software to extract the body regions from thermographic images^23^ that automatically recognize the player anatomy and segment the thermal image in body regions including 44 Regions Of Interest (ROIs) such as thighs, calves and hamstrings (see Fig. 1). Regions of interest were selected based on anatomical landmarks and predefined areas of interest. From those ROIs, average, minimum and maximal temperature were extracted to calculate thermal asymmetries between bilateral ROIs. Once the temperature values were extracted, statistical analyses were performed to explore potential thermal asymmetries and their relationships with physical demands and well-being. These analyses involved comparing temperature values between different muscle regions and assessing their associations with variables such as training load and wellness scores. Temperature was extracted to calculate thermal asymmetries between bilateral ROIs. Overall, the thermal image data analysis was a critical component of this study, as it provided valuable insights into the skin temperature variations and thermal asymmetries in elite football players.

The players were gathered into pairs in subgroups (low [< 0.3 °C] vs high thermal asymmetry [≥ 0.3 °C]) based on the results of the thermography session. Previous literature estimated clinically significant skin temperature asymmetry to exceed 0.5 °C^24^, but it was decided to establish the cut-off point at 0.3 °C because the sample was from a professional club and the members were highly supervised. The club's sports department decided by policy to track, take care, and monitor any player with an asymmetry of 0.3 °C or higher. Therefore, the sample was divided into low and high thermal asymmetry of all the analyzed muscle groups. The players were grouped according to the asymmetry of each one, for each muscle and each day. So, A player may therefore belong to a group with high asymmetry in one of the muscles under study and a group with low asymmetry in another muscle, and next days those muscles be in another group.

#### Movement patterns

Players wore GPS devices during matches and trainings for the 12 weeks period, the used devices were from WIMU PRO™ (RealTrack System SL, Almeria, Spain). The sample data rate was 18 Hz. These instruments were previously validated as a reliable gadget to collect physical information in team sports such as football^25^. Players wore a padded training vest under their shirt where the device was placed. Data was obtained and analyzed by the software SPRO™ v. 964 (Realtrack Systems S.L., Almería, Spain).

The analyzed variables were the following ones: Distance covered (m), High Speed Running (HSR: > 21.1 km/h) (m), Distance covered between 18 and 21 km/h (m), Distance covered between 21–24 km/h (m), Distance covered at > 24 km/h (m), Number of accelerations (≥ 3 m/s^2^) (n), Number of decelerations (≥ − 3 m/s^2^) (n), Distance covered accelerating (≥ 3 m/s^2^) (m), Distance covered decelerating (≥ − 3 m/s^2^) (m), Number of sprints (> 24 km/h) (n), High Metabolic Load Distance (HMLD) (m), Acute-Chronic HSR Load Coupled, Acute-Chronic HSR Load Uncoupled, Ratio Acute-Chronic HMLD Coupled, Ratio Acute-Chronic HMLD Uncoupled, Ratio Acute-Chronic Load at > 24 km/h Coupled, Ratio Acute-Chronic Load at > 24 km/h Uncoupled, Ratio Acute-Chronic Load distance accelerating Coupled, Ratio Acute-Chronic Load distance accelerating Uncoupled, Maximum speed (km/h) (V_max_), Maximum acceleration (m/s^2^) (ACC_max_) and Maximum deceleration (m/s^2^) (DEC_max_).

#### Wellness Questionnaire

Players were requested to fulfill a digital questionnaire on perceived well-being before every training session and match. The questionnaire was designed to be concise and easy to complete by the players. It was developed based on the questionnaires previously evaluated and validated in the existing literature^26^. In the provided questionnaire players had to answer their feeling from 1 to 10 about the following variables: (1) Modified version of Borg Rating of Perceived Exertion (RPE). This scale have been already validated^27^, and previous studies realized with football players have used this scale^8^. (2) Stress. (3) Rest time. (4) Rest quality. (5) Muscle Soreness. For RPE, stress and muscle soreness, a range of 1 was the best and 10 was the worst. For Rest time and Rest quality, the ranges were reversed, with 1 being the worst and 10 being the best.

### Statistical analysis

Means and standard deviations were presented for all quantitative variables. The Kolmogorov–Smirnov test and Levene test were used to confirm the normal distribution and the homogeneity of variance of the variables. Firstly, one way ANOVA with lineal and quadratic polynomic contrast was used in order to analyze the evolution of the main physical fitness variables during the season. After that, Independent Samples t Test was performed in order to compare the different physical demands between high thermal asymmetry (≥ 0.3 °C) and low thermal asymmetry (< 0.3 °C) groups for each muscle region. Furthermore, 95% confidence intervals and effect size differences (ES) using Cohen’s coefficient (Cohen 1992) were also calculated. The ES was interpreted as follows: < 0.2 = negligible; 0.2–0.6 = small; 0.6–1.2 = moderate; > 1.2 = large. Finally, Product–moment correlations (Pearson r) were performed to evaluate the relationship between thermal asymmetry and the physical demands. All data was statistically analyzed using SPSS Version 28.0 for Windows (IBM Corp, Chicago, IL).

## Results

**Figure 2:**
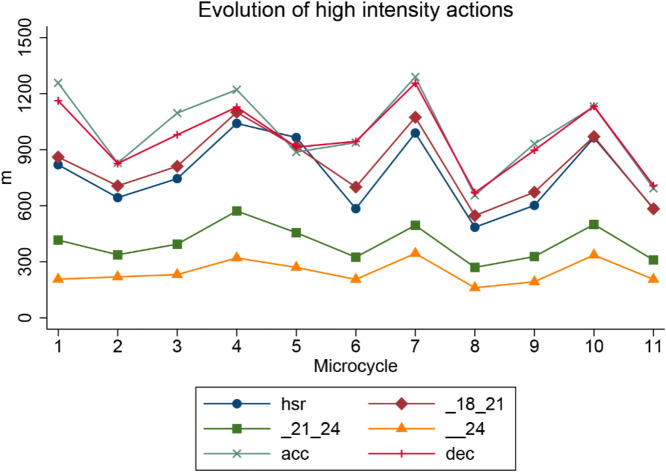
Evolution of High Intensity actions among the 11 competitive microcycles data collection.

Figure 2 shows the evolution of the high intensity actions among the 11 competitive microcycles that took place in the collection data process. The distance covered accelerating at high intensity (≥ 3 m/s^2^) was reduced from ~ 1250 m to 600 m. The distance covered decelerating at high intensity (≥ − 3 m/s^2^) was also reduced from ~ 1200 m to 600 m. The other variables were pretty consistent during the 11 competitive microcycles, but the four of them had a peak during microcycles 4, 7 and 10 of the data collection and in the following weeks went back to the initial values.

Tables 1 and 2 shows the differences in the external load and Wellness questionnaire variables for the asymmetry clusters. the thighs were divided in Outer Upper, Central Upper, Outer Front and Central Front. For the Outer Upper Thighs, there are significant differences with higher values for the high asymmetry group in the distance covered at HSR (m), distance covered between 18–21 km/h (m), distance covered > 24 km/h, distance covered accelerating (m), number of sprints and V_max_ (ES: 0.31–0.41). For the Central Upper thighs there were significant differences with higher values for the low asymmetry group for the Acute-Chronic workload distance covered over 24 km/h coupled and in V_max_ with higher values for the high asymmetry group (ES: 0.34–0.36). The Outer Front Thighs have significant differences with higher values for the high asymmetry group in total distance, number of accelerations, number of decelerations and muscle soreness (ES: 0.33–0.42). The Central Front thighs had significant differences with higher values for the low asymmetry group in DEC_max_ (ES: 0.40). For the adductor there were significant differences with higher values for the low asymmetry group in distance accelerating, also the high asymmetry group had statistically significant worse results in the Wellness variables: higher stress, higher muscle soreness and less rest (ES: 0.32–0.45). In the Knees the high asymmetry group had more fatigue, less rest and worse quality (ES: 0.35–0.41). For the ankle, there were significant differences with higher values for the low asymmetry group in distance in HSR, distance between 18 and 21 km/h, distance between 21 and 24 km/h, number of accelerations and distance in HMLD (m) (ES: 0.22–0.30). The hamstrings were divided into Central Back thigh and Outer Back thigh. The Central Back thigh had significant differences with higher values for low asymmetry group distance made at HSR, distance between 18 and 21 km/h, distance between 21 and 24 km/h, distance > 24 km/h, distance accelerating and number of sprints (ES: 0.32–0.42). Finally, in the Outer Back Leg there were significant differences with higher values for the low asymmetry group in ACC_max_ (p = 0.015; ES: 0.38; IC: − 0.47 to − 0.05).

**Table 1:** Differences in GPS and Wellness variables according to the asymmetry clusters.

Variable		Outer upper thigh asymmetry	Central upper thigh asymmetry	Outer front thigh asymmetry	Central front thigh asymmetry	Front adductor asymmetry	Inner front thigh asymmetry	Knee asymmetry	Ankle asymmetry
Distance (m)	HA	22,008.55 ± 6469.84	20,024.30 ± 6517.17	22,768.83 ± 6322.84	20,744.49 ± 6282.02	20,445.85 ± 7729.59	21,523.18 ± 5782.60	20,775.13 ± 6922.36	20,312.26 ± 7161.13
	LA	20,404.87 ± 7212.03	21,443.21 ± 7211.56	20,545.55 ± 7096.42	21,227.92 ± 7235.60	21,391.92 ± 6586.68	20,901.55 ± 7434.84	21,315.72 ± 7017.39	21,755.49 ± 6756.36
HSR (m)	HA	889.87* ± 446.22	752.36 ± 431.49	828.87 ± 425.03	746.53 ± 380.50	776.62 ± 416.74	733.20 ± 347.27	769.70 ± 432.50	706.50* ± 412.68
	LA	722.34* ± 425.04	792.47 ± 442.74	760.00 ± 442.45	788.94 ± 459.81	777.00 ± 449.38	795.95 ± 472.33	782.01 ± 444.01	837.03* ± 452.12
18–21 km/h (m)	HA	905.20* ± 417.98	779.10 ± 382.95	883.89 ± 369.21	810.01 ± 359.00	817.24 ± 403.19	848.14 ± 359.89	823.70 ± 407.57	766.31* ± 381.46
	LA	780.25* ± 372.73	847.87 ± 389.67	804.74 ± 392.26	829.75 ± 399.10	827.36 ± 381.16	813.66 ± 399.52	824.45 ± 373.99	873.56* ± 387.20
21–24 km/h (m)	HA	450.05 ± 221.61	392.80 ± 221.34	432.24 ± 215.82	389.12 ± 183.96	402.91 ± 218.32	399.88 ± 185.23	404.61 ± 230.17	372.37* ± 211.65
	LA	384.20 ± 217.98	414.10 ± 220.01	397.19 ± 222.09	412.40 ± 233.77	407.12 ± 222.38	408.35 ± 234.89	406.61 ± 214.42	434.33* ± 224.93
> 24 km/h (m)	HA	299.35* ± 201.58	248.60 ± 193.87	264.77 ± 185.47	240.96 ± 169.67	256.62 ± 177.05	214.26 ± 140.71	239.24 ± 177.02	220.24 ± 165.49
	LA	228.53* ± 175.60	252.94 ± 183.28	242.02 ± 185.20	250.24 ± 191.34	243.38 ± 189.18	262.15 ± 200.11	253.57 ± 191.13	270.98 ± 198.05
Acc (n)	HA	10,223.25 ± 2802.03	9316.92 ± 2875.58	10,530.88 ± 2223.81	9741.78 ± 2724.18	9397.57 ± 3295.98	9979.58 ± 2410.54	9716.26 ± 2826.30	9417.95 ± 2989.23
	LA	9527.93 ± 3060.91	10,013.56 ± 3043.23	9525.82 ± 3100.36	9784.23 ± 3026.37	9947.33 ± 2748.49	9681.60 ± 3142.97	9812.09 ± 3024.87	10,074.85 ± 2870.68
Dec (n)	HA	10,300.72 ± 2821.45	9386.48 ± 2901.19	10,603.82 ± 2241.35	9812.17 ± 2748.10	9460.92 ± 3317.21	10,050.77 ± 2428.62	9778.81 ± 2844.56	9476.27 ± 3004.82
	LA	9591.28 ± 3079.27	10,080.27 ± 3058.60	9591.33 ± 3119.50	9850.34 ± 3043.60	10,016.52 ± 2766.02	9747.24 ± 3162.25	9882.83 ± 3044.19	10,149.88 ± 2891.06
Acc (m)	HA	1087.68* ± 399.37	990.06 ± 432.14	1108.37 ± 397.71	983.58 ± 412.42	918.20* ± 441.82*	993.33 ± 400.57	1004.79 ± 428.37	935.12* ± 419.27
	LA	944.47* ± 429.84	983.40 ± 411.45	981.93 ± 435.92	1024.58 ± 436.79	1057.21* ± 417.72*	1021.48 ± 442.48	1018.73 ± 431.84	1079.41* ± 428.62
Dec (m)	HA	1037.77 ± 350.37	922.46 ± 376.02	1063.18 ± 363.48	958.77 ± 371.69	922.00 ± 414.09	980.24 ± 317.46	968.54 ± 373.74	930.18 ± 382.82
	LA	938.14 ± 394.40	995.18 ± 378.20	954.59 ± 384.37	990.12 ± 386.03	1008.89 ± 363.30	981.63 ± 407.16	990.25 ± 388.02	1024.81 ± 376.36
Sprint (n)	HA	20.22* ± 12.02	16.94 ± 11.81	18.12 ± 11.11	16.97 ± 10.95	16.94 ± 11.12	16.00 ± 9.68	16.78 ± 10.99	15.94 ± 11.38
	LA	16.00* ± 11.61	17.59 ± 11.90	16.94 ± 11.94	17.34 ± 12.05	17.37 ± 12.03	17.77 ± 12.51	17.55 ± 12.26	18.34 ± 11.95
HMLD (m)	HA	4064.54 ± 1525.93	3619.40 ± 1488.94	4152.84 ± 1475.08	3712.03 ± 1376.65	3726.92 ± 1651.70	3866.65 ± 1329.50	3785.10 ± 1606.75	3571.77* ± 1509.73
	LA	3652.00 ± 1527.64	3880.96 ± 1540.99	3706.07 ± 1535.91	3856.70 ± 1589.16	3857.03 ± 1473.76	3793.27 ± 1613.39	3837.34 ± 1478.91	4023.90* ± 1522.75
Ac-Cou HSR (m)	HA	0.97 ± 0.35	0.90 ± 0.41	1.02 ± 0.46	0.93 ± 0.36	1.00 ± 0.41	1.01 ± 0.41	0.99 ± 0.43	0.93 ± 0.43
	LA	0.97 ± 0.46	1.01 ± 0.42	0.99 ± 0.46	1.02 ± 0.49	0.99 ± 0.48	0.99 ± 0.48	1.00 ± 0.47	1.05 ± 0.48
Ac-Unc HSR (m)	HA	1.02 ± 0.53	0.96 ± 0.72	1.15 ± 0.89	0.96 ± 0.50	1.08 ± 0.63	1.11 ± 0.78	1.09 ± 0.77	0.98 ± 0.61
	LA	1.06 ± 0.85	1.09 ± 0.74	1.09 ± 0.93	1.17 ± 1.04	1.12 ± 1.03	1.11 ± 0.98	1.12 ± 1.01	1.22 ± 1.11
Ac- Cou HMLD (m)	HA	1.01 ± 0.27	1.01 ± 0.36	1.11 ± 0.39	0.98 ± 0.24	1.02 ± 0.26	1.08 ± 0.39	1.04 ± 0.34	1.00 ± 0.31
	LA	1.03 ± 0.34	1.03 ± 0.26	1.03 ± 0.34	1.07 ± 0.38	1.06 ± 0.39	1.03 ± 0.33	1.05 ± 0.36	1.08 ± 0.38
Ac-Unc HMLD (m)	HA	1.06 ± 0.43	1.10 ± 0.79	1.26 ± 0.95	1.00 ± 0.34	1.05 ± 0.37	1.25 ± 1.32	1.13 ± 0.75	1.05 ± 0.47
	LA	1.10 ± 0.70	1.05 ± 0.41	1.12 ± 0.92	1.21 ± 1.07	1.20 ± 1.09	1.11 ± 0.69	1.17 ± 1.04	1.24 ± 1.19
Ac- Cou > 24 km/h (m)	HA	0.91 ± 0.45	0.85* ± 0.59	1.04 ± 0.62	0.91 ± 0.54	1.01 ± 0.64	1.04 ± 0.61	1.01 ± 0.67	0.93 ± 0.59
	LA	1.00 ± 0.68	1.05* ± 0.61	1.00 ± 0.67	1.05 ± 0.69	1.01 ± 0.66	0.99 ± 0.68	1.01 ± 0.65	1.07 ± 0.71
Ac-Unc > 24 km/h (m)	HA	1.02 ± 0.94	0.85 ± 0.63	1.07 ± 0.73	1.05 ± 1.10	1.90 ± 6.43	1.32 ± 1.51	1.66 ± 5.63	1.08 ± 1.01
	LA	1.54 ± 4.80	1.71 ± 5.07	2.26 ± 10.35	2.34 ± 10.62	2.01 ± 9.99	2.27 ± 10.80	2.19 ± 10.80	2.75 ± 12.25
Ac- Cou Acc (m)	HA	1.03 ± 0.26	1.03 ± 0.36	1.12 ± 0.43	1.01 ± 0.26	1.03 ± 0.28	1.09 ± 0.40	1.08 ± 0.36	1.04 ± 0.32
	LA	1.05 ± 0.35	1.04 ± 0.27	1.05 ± 0.33	1.09 ± 0.39	1.08 ± 0.38	1.06 ± 0.34	1.06 ± 0.35	1.09 ± 0.38
Ac-Unc Acc (m)	HA	1.08 ± 0.42	1.14 ± 0.88	1.31 ± 1.10	1.03 ± 0.36	1.08 ± 0.41	1.25 ± 1.20	1.20 ± 0.87	1.10 ± 0.52
	LA	1.13 ± 0.78	1.07 ± 0.40	1.14 ± 0.83	1.24 ± 1.04	1.23 ± 1.06	1.15 ± 0.75	1.17 ± 0.94	1.25 ± 1.14
Vmax	HA	30.72* ± 2.29	30.58* ± 2.69	30.03 ± 2.57	30.17 ± 2.42	30.17 ± 2.13	29.95 ± 2.76	29.88 ± 2.40	29.84 ± 2.40
	LA	29.76* ± 2.32	29.71* ± 2.01	29.93 ± 2.36	29.87 ± 2.41	29.85 ± 2.53	29.96 ± 2.25	30.01 ± 2.43	30.05 ± 2.42
ACC_max_	HA	5.60 ± 0.58	5.61 ± 0.69	5.72 ± 0.80	5.69 ± 0.79	5.57 ± 0.71	5.49 ± 0.70	5.60 ± 0.73	5.54 ± 0.68
	LA	5.56 ± 0.73	5.56 ± 0.69	5.55 ± 0.70	5.54 ± 0.70	5.60 ± 0.74	5.63 ± 0.74	5.58 ± 0.73	5.63 ± 0.77
DEC_max_	HA	−4.81 ± 0.71	−4.75 ± 0.80	−4.90 ± 0.62	−4.67* ± 0.79	−4.81 ± 0.86	−4.74 ± 0.76	−4.90 ± 0.67	−4.87 ± 0.79
	LA	−4.85 ± 0.69	−4.89 ± 0.62	−4.87 ± 0.75	−4.96* ± 0.67	−4.91 ± 0.64	−4.93 ± 0.69	−4.85 ± 0.75	−4.88 ± 0.66
RPE	HA	3.56 ± 2.27	3.52 ± 1.96	4.00 ± 2.72	3.39 ± 2.00	3.84 ± 2.24	3.44 ± 2.09	4.22* ± 2.32	3.69 ± 2.22
	LA	3.53 ± 2.11	3.51 ± 2.24	3.52 ± 2.03	3.76 ± 2.32	3.56 ± 2.23	3.74 ± 2.29	3.29* ± 2.10	3.62 ± 2.25
Stress	HA	3.44 ± 1.38	3.45 ± 1.48	3.18 ± 1.37	3.33 ± 1.31	3.76* ± 1.39	3.13 ± 1.46	3.08 ± 1.47	3.31 ± 1.41
	LA	3.14 ± 1.42	3.05 ± 1.33	3.24 ± 1.39	3.18 ± 1.42	2.99* ± 1.32	3.27 ± 1.35	3.32 ± 1.32	3.16 ± 1.37
Rest	HA	7.10 ± 1.22	7.18 ± 1.32	7.00 ± 1.56	7.22 ± 1.43	6.82* ± 1.52	7.29 ± 1.35	6.95* ± 1.43	7.14 ± 1.17
	LA	7.35 ± 1.23	7.33 ± 1.13	7.31 ± 1.14	7.24 ± 1.19	7.41* ± 1.10	7.21 ± 1.23	7.41* ± 1.12	7.30 ± 1.33
Rest Quality	HA	6.56 ± 1.45	6.84 ± 1.46	6.50 ± 1.80	7.00 ± 1.51	6.61 ± 1.58	7.10 ± 1.50	6.46* ± 1.73	6.74 ± 1.49
	LA	7.00 ± 1.46	6.89 ± 1.46	6.91 ± 1.42	6.72 ± 1.54	6.89 ± 1.51	6.67 ± 1.54	7.02* ± 1.36	6.85 ± 1.57
Muscle Soreness	HA	3.90 ± 1.82	4.03 ± 1.71	4.45 ± 1.96	4.20 ± 1.71	4.29* ± 1.65	3.94 ± 1.85	4.12 ± 1.74	3.67 ± 1.56
	LA	3.92 ± 1.56	3.76 ± 1.56	3.72 ± 1.51	3.79 ± 1.63	3.74* ± 1.65	3.90 ± 1.58	3.78 ± 1.61	4.08 ± 1.72

Data are mean ± SD; *Significant differences between groups; HA = High Asymmetry; LA = Low Asymmetry; HSR = High Speed Running; Acc = Accelerations; Dec = Decelerations; HMLD = High Metabolic Load Distance; Ac- Cou HSR = Acute-Chronic HSR Load coupled; Ac- Unc HSR = Acute-Chronic HSR Uncoupled; Ac-Cou HMLD = Acute-Chronic HMLD Coupled; Ac-Unc HMLD = Acute-Chronic HMLD Uncoupled; Ac- Cou > 24 km/h = Acute-Chronic Load at > 24 km/h Coupled; Ac- Unc > 24 km/h = Acute-Chronic Load at > 24 km/h Uncoupled; Ac- Cou Acc = Acute-Chronic Load distance accelerating Coupled; Ac-Unc Acc = Acute-Chronic Load distance accelerating Uncoupled. Vmax = Maximum velocity; ACC_max_ = maximum acceleration; DEC_max_ = Maximum Deceleration.

**Table 2:** Differences in GPS and Wellness variables according to the asymmetry clusters.

Variable		Outer back thigh asymmetry	Central back thigh asymmetry	Back adductor asymmetry	Inner back thigh asymmetry	Outer back leg asymmetry	Inner back leg asymmetry
Distance (m)	HA	21,577.18 ± 6959.16	20,571.91 ± 6723.00	21,252.55 ± 6595.40	21,933.55 ± 6476.09	21,792.45 ± 6582.31	21,142.33 ± 6529.77
	LA	20,977.55 ± 6979.01	21,490.52 ± 7063.63	21,192.55 ± 7223.75	20,824.82 ± 7204.83	20,858.19 ± 7187.91	21,285.47 ± 7365.13
HSR (m)	HA	768.49 ± 408.65	665.00* ± 394.02	799.60 ± 471.00	823.31 ± 458.13	816.70 ± 412.42	796.34 ± 457.72
	LA	791.51 ± 457.59	832.13* ± 447.19	770.61 ± 415.44	759.91 ± 426.43	760.91 ± 453.24	769.40 ± 420.42
18–21 km/h (m)	HA	847.25 ± 389.68	744.22* ± 378.50	813.96 ± 382.27	875.84 ± 384.64	858.84 ± 357.11	826.72 ± 359.58
	LA	819.83 ± 384.23	867.50* ± 384.12	842.16 ± 389.15	806.12 ± 385.48	813.28 ± 402.86	834.50 ± 409.96
21–24 km/h (m)	HA	410.28 ± 213.16	345.63* ± 196.91	409.06 ± 230.23	427.12 ± 231.88	424.48 ± 207.40	411.99 ± 226.06
	LA	408.44 ± 226.70	436.15* ± 225.55	409.26 ± 215.25	399.36 ± 214.88	399.64 ± 229.15	406.58 ± 217.00
> 24 km/h (m)	HA	244.13 ± 175.05	203.70* ± 173.10	254.81 ± 193.70	267.66 ± 180.49	258.35 ± 168.20	258.38 ± 194.43
	LA	253.23 ± 193.02	269.08* ± 187.97	246.07 ± 180.76	239.72 ± 188.39	244.15 ± 196.23	241.51 ± 177.75
Acc (n)	HA	9862.18 ± 2873.53	9495.44 ± 2682.38	10,044.90 ± 2768.90	10,047.76 ± 2726.29	9971.17 ± 2914.68	9686.45 ± 2824.40
	LA	9754.76 ± 2987.88	9925.88 ± 3037.27	9629.97 ± 3044.05	9660.82 ± 3046.12	9689.50 ± 2955.84	9900.15 ± 3045.28
Dec (n)	HA	9930.97 ± 2890.21	9556.22 ± 2701.62	10,110.60 ± 2788.00	10,115.61 ± 2743.77	10,036.63 ± 2934.42	9753.98 ± 2839.96
	LA	9821.50 ± 3007.69	9996.32 ± 3055.32	9698.79 ± 3062.64	9728.22 ± 3065.35	9758.37 ± 2973.91	9967.74 ± 3066.55
Acc (m)	HA	1040.49 ± 449.27	913.82* ± 395.02	1078.48 ± 459.71	1048.06 ± 432.14	1014.81 ± 409.12	1043.90 ± 430.17
	LA	1003.58 ± 419.34	1062.65* ± 438.97	977.51 ± 406.97	1002.03 ± 430.87	1020.48 ± 445.40	994.69 ± 432.11
Dec (m)	HA	982.37 ± 366.82	910.92 ± 377.36	1023.74 ± 419.12	1026.31 ± 358.05	999.62 ± 356.64	991.93 ± 338.54
	LA	988.84 ± 390.54	1018.24 ± 378.39	960.84 ± 351.18	964.35 ± 391.60	977.93 ± 395.57	981.03 ± 416.75
Sprint (n)	HA	17.01 ± 10.95	13.85* ± 9.73	17.99 ± 12.88	17.94 ± 11.47	18.25 ± 10.80	17.62 ± 11.80
	LA	17.55 ± 12.37	18.81* ± 12.31	16.89 ± 11.04	17.00 ± 12.01	16.76 ± 12.39	17.07 ± 11.85
HMLD (m)	HA	3905.53 ± 1497.47	3536.94 ± 1465.84	3826.04 ± 1518.04	4023.51 ± 1462.59	3921.56 ± 1409.44	3838.26 ± 1422.49
	LA	3802.98 ± 1549.71	3974.19 ± 1537.58	3856.01 ± 1537.85	3745.67 ± 1556.54	3795.52 ± 1598.19	3849.09 ± 1622.70
Ac- Cou HSR (m)	HA	1.02 ± 0.49	0.95 ± 0.49	0.97 ± 0.50	1.01 ± 0.49	0.96 ± 0.37	0.98 ± 0.40
	LA	0.96 ± 0.42	1.00 ± 0.43	0.99 ± 0.42	0.97 ± 0.42	1.00 ± 0.49	0.99 ± 0.49
Ac-Unc HSR (m)	HA	1.13 ± 0.86	1.08 ± 1.05	1.11 ± 1.08	1.18 ± 1.13	1.01 ± 0.51	1.06 ± 0.72
	LA	1.06 ± 0.89	1.09 ± 0.80	1.07 ± 0.72	1.04 ± 0.70	1.14 ± 1.04	1.11 ± 1.00
Ac- Cou HMLD (m)	HA	1.07 ± 0.39	1.00 ± 0.29	1.03 ± 0.39	1.05 ± 0.39	1.00 ± 0.27	1.03 ± 0.33
	LA	1.01 ± 0.28	1.05 ± 0.34	1.04 ± 0.28	1.02 ± 0.29	1.05 ± 0.36	1.04 ± 0.33
Ac- Unc HMLD (m)	HA	1.23 ± 1.22	1.04 ± 0.46	1.17 ± 1.23	1.21 ± 1.29	1.03 ± 0.37	1.14 ± 1.08
	LA	1.04 ± 0.44	1.15 ± 0.97	1.08 ± 0.45	1.07 ± 0.46	1.17 ± 1.04	1.10 ± 0.56
Ac- Cou > 24 km/h (m)	HA	1.04 ± 0.68	0.95 ± 0.76	0.98 ± 0.69	1.05 ± 0.74	0.95 ± 0.53	1.00 ± 0.61
	LA	0.95 ± 0.59	1.00 ± 0.56	0.99 ± 0.59	0.95 ± 0.56	1.01 ± 0.68	0.97 ± 0.64
Ac- Unc > 24 km/h (m)	HA	1.88 ± 5.83	3.75 ± 16.43	2.65 ± 13.21	3.58 ± 15.21	1.09 ± 1.03	1.68 ± 5.34
	LA	2.06 ± 10.83	1.22 ± 1.29	1.54 ± 4.78	1.11 ± 1.05	2.55 ± 11.58	2.28 ± 11.61
Ac-Cou Acc (m)	HA	1.08 ± 0.41	1.03 ± 0.29	1.07 ± 0.39	1.06 ± 0.39	1.02 ± 0.29	1.06 ± 0.34
	LA	1.04 ± 0.26	1.06 ± 0.35	1.05 ± 0.28	1.05 ± 0.29	1.08 ± 0.35	1.05 ± 0.32
Ac- Unc Acc (m)	HA	1.25 ± 1.15	1.08 ± 0.45	1.21 ± 1.14	1.22 ± 1.19	1.07 ± 0.42	1.17 ± 0.99
	LA	1.07 ± 0.39	1.17 ± 0.90	1.09 ± 0.44	1.10 ± 0.45	1.19 ± 0.95	1.11 ± 0.55
Vmax	HA	30.39 ± 2.37	29.60 ± 2.08	29.95 ± 2.51	30.16 ± 2.40	30.02 ± 2.37	30.14 ± 2.19
	LA	29.72 ± 2.33	30.16 ± 2.46	30.02 ± 2.27	29.90 ± 2.35	29.97 ± 2.37	29.85 ± 2.52
ACC_max_	HA	5.60 ± 0.67	5.63 ± 0.72	5.67 ± 0.65	5.63 ± 0.66	5.44* ± 0.54	5.56 ± 0.61
	LA	5.61 ± 0.77	5.59 ± 0.74	5.56 ± 0.79	5.59 ± 0.77	5.70* ± 0.81	5.64 ± 0.83
DEC_max_	HA	−4.81 ± 0.63	−4.84 ± 0.75	−4.95 ± 0.66	−4.88 ± 0.55	−4.85 ± 0.65	−4.91 ± 0.68
	LA	−4.93 ± 0.78	−4.90 ± 0.72	−4.83 ± 0.76	−4.88 ± 0.81	−4.90 ± 0.77	−4.86 ± 0.76
RPE	HA	3.49 ± 2.29	4.09 ± 2.31	3.60 ± 2.30	3.74 ± 2.59	3.38 ± 2.23	3.35 ± 2.24
	LA	3.75 ± 2.23	3.47 ± 2.21	3.67 ± 2.23	3.58 ± 2.03	3.82 ± 2.26	3.94 ± 2.24
Stress	HA	3.07 ± 1.37	3.04 ± 1.30	2.99 ± 1.26	3.11 ± 1.34	3.11 ± 1.36	3.14 ± 1.18
	LA	3.28 ± 1.33	3.26 ± 1.37	3.35 ± 1.40	3.25 ± 1.36	3.26 ± 1.35	3.26 ± 1.51
Descanso	HA	7.18 ± 1.31	7.13 ± 1.09	7.28 ± 1.35	7.31 ± 1.06	7.31 ± 1.21	7.32 ± 1.12
	LA	7.22 ± 1.26	7.23 ± 1.35	7.15 ± 1.23	7.14 ± 1.39	7.13 ± 1.32	7.09 ± 1.42
Rest Quality	HA	6.85 ± 1.59	6.70 ± 1.40	6.73 ± 1.64	6.66 ± 1.52	6.83 ± 1.59	6.69 ± 1.41
	LA	6.73 ± 1.53	6.81 ± 1.61	6.81 ± 1.49	6.85 ± 1.57	6.74 ± 1.53	6.86 ± 1.68
Muscle Soreness	HA	3.93 ± 1.80	3.83 ± 1.72	3.64 ± 1.64	3.80 ± 1.87	4.09 ± 1.72	3.84 ± 1.62
	LA	3.94 ± 1.60	3.97 ± 1.67	4.14 ± 1.69	4.01 ± 1.56	3.83 ± 1.66	4.02 ± 1.75

Data are mean ± SD; *Significant differences between groups; HA = High Asymmetry; LA = Low Asymmetry; HSR = High Speed Running; Acc = Accelerations; Dec = Decelerations; HMLD = High Metabolic Load Distance; Ac- Cou HSR = Acute-Chronic HSR Load coupled; Ac- Unc HSR = Acute-Chronic HSR Uncoupled; Ac-Cou HMLD = Acute-Chronic HMLD Coupled; Ac-Unc HMLD = Acute-Chronic HMLD Uncoupled; Ac- Cou > 24 km/h = Acute-Chronic Load at > 24 km/h Coupled; Ac- Unc > 24 km/h = Acute-Chronic Load at > 24 km/h Uncoupled; Ac- Cou Acc = Acute-Chronic Load distance accelerating Coupled; Ac-Unc Acc = Acute-Chronic Load distance accelerating Uncoupled. Vmax = Maximum velocity; ACC_max_ = maximum acceleration; DEC_max_ = Maximum Deceleration.

Table 3 shows the correlations between the asymmetry of the thermograms of selected ROIs and GPS and Wellness variables. It can be noted that there are positive correlations between high-intensity actions and thermal asymmetry of the thigh muscles, and negative correlations with the hamstring’s muscles. Acute-chronic workloads have positive correlations with the thermal asymmetry of the hamstrings and negative for the thigh’s muscles. Higher thermal asymmetries are associated with worse wellness results, except for the Global Body Assessment (TRI).

**Table 3:** Correlation between thermal asymmetries with GPS and Wellness variables.

	TRI	Outer upper thigh asymmetry	Central upper thigh asymmetry	Outer front thigh asymmetry	Central front thigh asymmetry	Front adductor asymmetry	Inner front thigh asymmetry	Outer back thigh asymmetry	Central back thigh asymmetry	Back adductor asymmetry	Inner back thigh asymmetry	Outer back leg asymmetry	Inner back leg asymmetry	Knee asymmetry	Ankle asymmetry
Distance (m)	−0.021	0.126	−0.102	0.084	−0.094	−0.059	0.000	0.043	−0.037	0.018	0.026	0.046	−0.014	−0.033	−0.109
HSR (m)	0.020	0.172*	−0.030	0.050	−0.122	−0.028	−0.100	0.001	−0.144*	0.077	0.023	0.056	−0.012	−0.014	−0.152*
18–21 km/h (m)	0.003	0.157*	−0.086	0.044	−0.074	−0.037	−0.001	0.048	−0.127	0.010	0.010	0.034	−0.039	−0.030	−0.132
21–24 km/h (m)	−0.003	0.150*	−0.032	0.037	−0.106	−0.034	−0.071	0.029	−0.180*	0.036	−0.005	0.047	−0.021	−0.037	−0.141*
> 24 km/h (m)	0.022	0.155*	0.018	0.025	−0.110	0.027	−0.132	−0.014	−0.123	0.062	0.052	0.048	−0.011	−0.012	−0.149*
Acc (n)	−0.004	0.126	−0.104	0.099	−0.092	−0.053	0.034	0.025	0.000	0.043	0.059	0.023	−0.009	0.004	−0.130
Dec (n)	−0.006	0.128	−0.103	0.100	−0.092	−0.053	0.034	0.025	−0.002	0.042	0.058	0.022	−0.010	0.002	−0.132
Acc (m)	0.009	0.147*	−0.015	0.090	−0.095	−0.139*	−0.026	0.045	−0.094	0.105	0.007	0.038	0.081	−0.017	−0.233**
Dec (m)	0.024	0.119	−0.113	0.060	−0.101	−0.098	−0.006	0.025	−0.055	0.097	0.086	0.070	0.041	0.001	−0.145*
Sprint (n)	0.039	0.135	0.000	0.029	−0.116	−0.012	−0.103	0.010	−0.175*	0.063	−0.012	0.053	−0.003	−0.034	−0.123
HMLD (m)	−0.020	0.143	−0.091	0.068	−0.100	−0.056	−0.019	0.040	−0.099	0.018	0.043	0.035	−0.020	−0.024	−0.149*
Ac-Ac HSR (m)	−0.067	0.016	−0.174*	0.042	−0.115	0.044	−0.039	0.075	−0.058	−0.044	0.033	−0.025	−0.042	−0.006	−0.109
Ac-De HSR (m)	−0.052	−0.007	−0.133	0.049	−0.125	0.011	−0.064	0.062	0.008	0.019	0.074	−0.045	−0.046	0.000	−0.132
Ac-Ac HMLD (m)	−0.076	0.016	−0.115	0.117	−0.159*	0.017	−0.015	0.104	−0.071	−0.051	0.010	−0.066	−0.009	−0.004	−0.107
Ac-De HMLD (m)	−0.016	0.018	−0.043	0.097	−0.117	0.006	−0.014	0.139	−0.060	0.034	0.042	−0.052	0.039	0.008	−0.121
Ac-Ac > 24 km/h (m)	−0.024	−0.071	−0.187*	0.023	−0.119	0.022	−0.021	0.077	0.002	−0.021	0.086	−0.029	−0.065	0.028	−0.098
Ac-De > 24 km/h (m)	−0.008	−0.080	−0.119	−0.016	−0.066	−0.038	−0.085	−0.050	0.176*	0.071	0.148*	−0.065	−0.054	0.013	−0.105
Ac-Ac Acc (m)	−0.048	0.022	−0.092	0.087	−0.149*	0.020	−0.050	0.091	−0.058	−0.047	−0.003	−0.086	0.014	0.040	−0.097
Ac-De Acc (m)	0.009	0.015	−0.017	0.100	−0.118	0.015	−0.042	0.144*	−0.053	0.029	0.036	−0.063	0.048	0.056	−0.116
Vmax	0.099	0.172*	0.182*	−0.020	−0.013	0.080	−0.007	0.125	−0.158*	−0.009	−0.032	−0.025	0.024	−0.108	−0.073
ACC_max_	−0.039	0.038	0.030	0.019	0.069	0.059	0.049	−0.037	0.042	0.080	0.040	−0.147*	−0.047	0.071	−0.105
DEC_max_	−0.017	0.071	0.080	0.011	0.108	0.033	0.110	0.063	−0.022	−0.133	−0.071	−0.066	−0.032	−0.062	−0.005
RPE	−0.033	0.012	0.014	0.069	−0.181*	0.054	−0.089	−0.079	0.140	−0.036	0.120	−0.077	−0.145	0.242**	−0.036
Stress	−0.158*	0.074	0.083	0.086	−0.024	0.260**	−0.030	−0.096	−0.144	−0.123	−0.099	−0.063	−0.043	−0.132	0.058
Rest	−0.106	−0.131	−0.112	−0.023	0.003	−0.181*	0.014	0.004	−0.067	0.142	−0.050	0.093	0.088	−0.180*	−0.108
Rest Quality	−0.053	−0.172*	−0.022	−0.019	0.101	−0.069	0.140	0.069	−0.064	0.006	−0.133	−0.010	−0.027	−0.133	−0.081
Muscle Soreness	−0.095	0.025	0.075	0.234**	0.041	0.174*	−0.069	−0.063	−0.094	−0.123	0.003	0.072	−0.040	0.073	−0.142

Data are mean ± SD; *Correlation statistically significative in 0.01 level; **Correlation statistically significative in 0.05 level; TRI = Thermal Risk Index; HSR = High Speed Running; Acc = Accelerations; Dec = Decelerations; HMLD = High Metabolic Load Distance; Ac- Cou HSR = Acute-Chronic HSR Load Uncoupled; Ac- Unc HSR = Acute-Chronic HSR Uncoupled; Ac-Cou HMLD = Acute-Chronic HMLD Coupled; Ac-Unc HMLD = Acute-Chronic HMLD Uncoupled; Ac- Cou > 24 km/h = Acute-Chronic Load at > 24 km/h Coupled; Ac- Unc > 24 km/h = Acute-Chronic Load at > 24 km/h Uncoupled; Ac- Cou Acc = Acute-Chronic Load distance accelerating Coupled; Ac-Unc Acc = Acute-Chronic Load distance accelerating Uncoupled. Vmax = Maximum velocity; ACC_max_ = maximum acceleration; DEC_max_ = Maximum Deceleration.

Table 4 displays the correlations between the mean temperature of the thermograms of selected ROIs and GPS and Wellness variables. It can be highlighted that the ankle temperature is positively correlated with high-intensity actions. Acute-chronic workload is negatively correlated with the mean temperature of the thigh and hamstrings muscles, and the wellness results do not show a clear trend, with both positive and negative correlations for the different muscles and wellness variables.

**Table 4:** Correlation between mean skin temperatures with GPS and Wellness variables.

	TRI	Outer upper thigh mean temperature	Central upper thigh mean temperature	Outer front thigh mean temperature	Central front thigh mean temperature	Front adductor mean temperature	Inner front thigh mean temperature	Outer back thigh mean temperature	Central back thigh mean temperature	Back adductor mean temperature	Inner back thigh mean temperature	Outer back leg mean temperature	Inner back leg mean temperature	Knee mean temperature	Ankle mean temperature
Distance (m)	−0.021	0.017	−0.038	−0.011	−0.054	−0.056	−0.036	−0.072	−0.074	−0.048	−0.080	−0.069	−0.072	−0.045	0.004
HSR (m)	0.020	0.045	0.013	0.024	−0.016	−0.001	−0.016	−0.049	−0.105	−0.081	−0.083	−0.016	−0.023	−0.023	0.123
18–21 km/h (m)	0.003	0.021	−0.024	−0.014	−0.044	−0.061	−0.049	−0.070	−0.076	−0.066	−0.079	−0.057	−0.088	−0.081	0.035
21–24 km/h (m)	−0.003	0.066	0.031	0.032	−0.006	0.011	−0.012	−0.037	−0.066	−0.055	−0.047	0.001	−0.017	−0.042	0.111
> 24 km/h (m)	0.022	0.072	0.035	0.060	0.016	0.044	0.023	−0.019	−0.072	−0.040	−0.053	0.017	0.036	0.049	0.188**
Acc (n)	−0.004	−0.033	−0.070	−0.018	−0.054	−0.056	−0.025	−0.022	−0.073	−0.046	−0.076	−0.049	−0.034	0.023	0.092
Dec (n)	−0.006	−0.033	−0.070	−0.019	−0.054	−0.056	−0.026	−0.023	−0.073	−0.046	−0.076	−0.050	−0.035	0.023	0.093
Acc (m)	0.009	0.054	0.017	0.036	0.002	0.020	0.007	0.016	−0.081	−0.031	−0.047	−0.038	−0.049	−0.057	0.054
Dec (m)	0.024	0.083	0.048	0.052	0.036	0.025	0.048	0.060	−0.025	0.001	−0.008	0.002	−0.006	−0.014	0.066
Sprint (n)	0.039	−0.003	−0.022	−0.010	−0.052	−0.011	−0.054	−0.075	−0.134	−0.101	−0.112	−0.047	−0.036	−0.053	0.137
HMLD (m)	−0.020	0.052	−0.008	0.012	−0.026	−0.043	−0.019	−0.040	−0.049	−0.028	−0.061	−0.051	−0.071	−0.051	0.041
Ac-Ac HSR (m)	−0.067	−0.134	−0.126	−0.118	−0.125	−0.116	−0.132	−0.078	−0.102	−0.107	−0.136	−0.111	−0.110	−0.094	−0.075
Ac-De HSR (m)	−0.052	−0.149*	−0.129	−0.110	−0.109	−0.105	−0.111	−0.074	−0.108	−0.123	−0.124	−0.090	−0.091	−0.080	−0.096
Ac-Ac HMLD (m)	−0.076	−0.213**	−0.211**	−0.155*	−0.150*	−0.158*	−0.152*	−0.058	−0.083	−0.082	−0.111	−0.090	−0.094	−0.129	−0.077
Ac-De HMLD (m)	−0.016	−0.242**	−0.236**	−0.113	−0.112	−0.120	−0.105	−0.015	−0.059	−0.063	−0.067	−0.031	−0.044	−0.116	−0.088
Ac-Ac > 24 km/h (m)	−0.024	−0.103	−0.103	−0.064	−0.071	−0.072	−0.053	−0.026	−0.066	−0.080	−0.086	−0.034	−0.037	−0.026	−0.074
Ac-De > 24 km/h (m)	−0.008	0.101	0.088	0.021	0.024	−0.015	0.026	−0.051	−0.065	−0.101	−0.058	−0.030	−0.018	0.028	−0.024
Ac-Ac Acc (m)	−0.048	−0.206**	−0.225**	−0.143*	−0.147*	−0.179*	−0.143*	−0.063	−0.070	−0.063	−0.088	−0.071	−0.072	−0.084	−0.041
Ac-De Acc (m)	0.009	−0.231**	−0.249**	−0.123	−0.128	−0.155*	−0.118	−0.023	−0.056	−0.056	−0.059	−0.029	−0.038	−0.098	−0.080
Vmax	0.099	0.026	−0.033	0.058	0.014	−0.003	0.023	−0.044	−0.052	−0.076	0.002	0.047	0.051	0.051	0.247**
ACC_max_	−0.039	0.115	0.081	0.108	0.094	0.043	0.059	0.073	−0.003	0.040	0.024	0.034	−0.003	0.084	0.142*
DECmax	−0.017	−0.102	−0.129	−0.107	−0.122	−0.118	−0.110	−0.086	−0.028	−0.067	−0.058	−0.068	−0.027	−0.010	0.038
RPE	−0.033	−0.037	−0.047	0.065	0.018	−0.033	0.061	0.014	0.002	0.004	0.056	0.029	0.023	0.209**	0.116
Stress	−0.158*	−0.141	−0.142	−0.073	−0.126	−0.161*	−0.186*	−0.143	−0.059	−0.075	−0.080	−0.087	−0.090	0.075	0.153*
Descanso	−0.106	−0.025	0.001	−0.110	−0.070	−0.007	−0.116	−0.097	−0.110	−0.059	−0.098	−0.119	−0.202**	−0.201**	−0.127
Rest Quality	−0.053	−0.003	0.027	−0.099	−0.060	−0.049	−0.033	0.021	0.021	−0.007	0.009	−0.017	−0.077	−0.089	−0.079
Muscle soreness	−0.095	−0.230**	−0.217**	−0.095	−0.148	−0.129	−0.116	−0.144	−0.073	−0.143	−0.071	−0.027	0.003	0.085	0.146

Data are mean ± SD; *Correlation statistically significative in 0.01 level; **Correlation statistically significative in 0.05 level; TRI = Thermal Risk Index; HSR = High Speed Running; Acc = Accelerations; Dec = Decelerations; HMLD = High Metabolic Load Distance; Ac- Cou HSR = Acute-Chronic HSR Load Uncoupled; Ac- Unc HSR = Acute-Chronic HSR Uncoupled; Ac-Cou HMLD = Acute-Chronic HMLD Coupled; Ac-Unc HMLD = Acute-Chronic HMLD Uncoupled; Ac- Cou > 24 km/h = Acute-Chronic Load at > 24 km/h Coupled; Ac- Unc > 24 km/h = Acute-Chronic Load at > 24 km/h Uncoupled; Ac- Cou Acc = Acute-Chronic Load distance accelerating Coupled; Ac-Unc Acc = Acute-Chronic Load distance accelerating Uncoupled. Vmax = Maximum velocity; ACC_max_ = maximum acceleration; DEC_max_ = Maximum Deceleration.

## Discussion

The purpose of this study was to check the association between physical demands, skin temperature and wellbeing status in elite football players. Thereby, during 11 competitive microcycles throughout 21/22 season (14th February–11st May) 30 players underwent a thermography assessment three days after the last match, fulfilled a wellness questionary weekly and in addition their match and training external load with GPS was collected. Thermography provides information about the skin temperature of the analyzed body regions. The variation in skin temperature is related to physiological processes that occur in response to training stimuli^28^. Additionally, thermal asymmetries between body hemispheres have shown their utility in identifying injury risk markers^15,16^. The body maintains a homeostatic relationship with its temperature, so healthy athletes tend to have an asymmetry of less than 0.3 °C^29^. Therefore, if a situation of hyperthermia, more related to increased metabolic demand^30^, or hypothermia, more related to damage to the nervous system^31^, occurs, it should be taken into consideration.

About the results, it can be resumed that High-intensity actions contribute to develop more asymmetries in the thighs. Higher hamstring asymmetries make it harder for players to execute all the high-intensity tasks required to compete at a high level, while adductor asymmetries are linked to worse recovery and soreness scores. It has also been found that there is a relationship between muscular asymmetry and wellbeing, with higher asymmetries producing worse wellbeing. It is also important to take into account the mean temperature because it may affect muscle function and recovery, although in different ways depending on the muscle.

The distance covered accelerating at high intensity (≥ 3 m/s^2^) and decelerating at high intensity (≥ − 3 m/s^2^) along the first microcycle to the last one. These actions significantly contribute to muscle damage post-match^32^ and were found to decrease in professional team sports from first to second half, suggesting an association with muscle neuromuscular fatigue^3^, moreover, there was also found a positive correlation between RPE and accelerations, when accelerations increase the RPE did^33^. In this particular case, the accelerations and decelerations decreasement might be the consequence of a training loads reduction by the team prior to face the final matches of the season and the play offs, the players were fatigated because of the competition demands, so training loads were reduced for players to be optimal for those important matches. On the other hand, the reason why the rest of high intensity actions (HSR (m); 18–21 km/h (m); 21–24 km/h (m); > 24 km/h (m)) were not reduced can be attributable to a maintenance of the intensity, as maintaining the intensity and performing these actions during training can be crucial to maintain physical condition; high-speed running actions and sprints, are considered a prerequisite for successful performance in football^34^, besides, straight sprints are the movements most commonly performed when scoring a goal or dodging an opponent^35^. So, following the tapering principles of the literature, total load was reduced and intensity was maintained^36^. In this sense training loads were high enough to allow players to complete distances at high intensity but not sufficient to let them reach accelerations and deceleration previous distances values.

Concerning the thighs ROI, the Outer Upper Thighs, high asymmetry group had higher values with significant differences in the distance covered at HSR, distance covered over 24 km/h, distance covered accelerating, number of sprints and V_max_. The central Upper thighs had higher values in the high asymmetry group for V_max_. And the Outer Front Thighs high asymmetry group had higher values with significant differences for Accelerations (n), Decelerations (n) and muscle soreness values. The results can lead to the conclusion that high intensity actions significantly contribute to generate thermal asymmetries in the thighs, so players that perform more of these actions generate greater asymmetries. These results are related with previous studies of the literature, that found that after aerobic exercise (running 1 h at 60% VO^2^_max_) temperature of the thighs was higher than at the beginning (while temperature of the upper limbs was lower than at the beginning)^37^; moreover Merla et al.,^38^ found that when athletes exercise until maximum heart rate, temperature increase in the recovery phase post-exercise, and Fernandes et al.,^39^ concluded the same with interval running in the treadmill. Thereby, if running, and specially interval running increase the temperature, it is possible that it enlarge the thermal asymmetries, especially in an intermittent and unpredictable sport like football, as the intermittent running can increase temperature and the uncontrolled situations such as kicking or tackling can produce and enlarge asymmetries. The analogous observations between our study and^37–39^ researches highlight the potential impact of exercise intensity and duration on skin temperature in athletic contexts. However, it's important to consider the specific demands of football, such as sprinting, direction changes, and body contact, which may further contribute to thermal asymmetries in specific muscle groups over running. Even though, more investigation is needed to corroborate these findings and understand the dynamic relationship between physical demands of football and skin temperature football players. Moreover, the only wellness variable influenced by thigh asymmetry was the muscle soreness, relating this with the preceding explanation, if the players with higher asymmetries in the thighs are the ones who run more and perform more high intensity actions, it is expected that those athletes feel more muscle soreness. On the other hand, for Central Front Thighs DEC_max_, the low asymmetry group had higher values, but effect size is not very high, so that difference can be due to one or two players of the high asymmetry group reducing markedly the load to prevent injuries.

For the Adductor ROI, in the high asymmetry group, there is a positive relationship with the stress and muscular soreness items, which indicates that the higher the asymmetry, the worse the perception of recovery. In addition, the resting item has a negative relationship, which indicates that the better the rest, the less asymmetry. This region may be one of the most responsive to competition load (moreover, high asymmetry group of this muscle completed more distance accelerating) due to its relationship with core temperature, and the type of fatigue generated. With higher the skin temperatures response, there is more fatigue generated, so it takes longer to recover^40^.

Regarding the ROI of the knees, there is a positive relationship between high thermal asymmetries and fatigue items, and a negative relationship with respect to rest and quality of rest. This could be due to the fact that, as has been seen in previous research, an increase in thermal imbalances may have mechanical causes due to functional overload^41^. This could indicate that those who generate more asymmetries carried out higher mechanical loads and accumulated more fatigue, as a consequence they are resting less time and with worse quality, circumstances that did not allow them to recover properly from the training and matches loads.

In respect to the hamstring ROI, the Central Back of the hamstrings have significant differences with higher values for the low asymmetry group in the distance made at HSR, between 18–21 km/h, between 21 and 24 km/h, > 24 km/h, accelerating and sprinting, so players with low asymmetry in hamstring performed more of these actions, the reason could be that sprinting involves and heavily relies on the hamstrings, which are essential for generating force during the sprint's propulsive phase^42^ and running speed increases of 80–100% are associated with additions in net hamstring muscle force and energy absorption of 1.4 and 1.9 times respectively^43^. In this sense, players with high asymmetry of these muscles were not able to complete as many sprints and high intensity actions as the low asymmetry group, maybe for the lack of capacity, as hamstrings thermal asymmetries seem to have influence in sprint performance^44^ and are markedly the most frequently injured muscle during sprinting^45^.

For the ankle, similarly to hamstrings there were significant differences with higher values for the low asymmetry group in distance in HSR, between 18 and 21 km/h, 21–24 km/h, distance accelerating, and distance in HMLD. So, it looks that higher values of the ankle asymmetry reduce the acceleration capacity, maybe lower asymmetry in the ankle could result in a more balanced and stable gait, allowing for better transfer of energy and less impact on the joints during running, allowing positive impact on the players' performance in terms of running distance and speed, although more investigation is needed to confirm this statement. Finally, for the last significant difference, the low asymmetry group of the Inner back leg had higher values in the maximum acceleration (ACC_max_) than the high asymmetry group, so similarly to ankles, a reduced asymmetry of this muscle may lead to a smoother and more stable running style, leading to greater energy transfer and reduced joint stress during running. This, in turn, can enhance acceleration performance. Even though, specific investigations are needed to confirm this hypothesis.

Regarding the asymmetry correlations (Table 3), for workload variables there is a negative correlation statistically significant between Hamstrings thermal asymmetries and High Intensity Actions (HSR, distance between 21 and 24 km/h, number of sprints). These correlations are in line with the previous justifications; players with high asymmetry in hamstring realized less high intensity actions, maybe because these muscle is crucial for these actions^42,44^. Thereby, they lack from the capacity to perform them or perhaps to protect theirselves from an injury as hamstring are the most commonly injury muscle with the high intensity actions in football^45^. Moreover, similar to hamstrings, there is a negative correlation between the Ankle asymmetry and HSR, distance covered between 21 and 24 km/h, > 24 km/h, distance accelerating and decelerating, and HMLD, so, as commented in the external load results, it looks that higher values of the ankle asymmetry reduce the acceleration capacity. Also, there is negative correlation between adductor asymmetry and distance accelerating, maybe due to muscle imbalances, decreased strength in the adductor muscles, or altered biomechanics during movement. It's important to note that a correlation does not imply causality, so further research is needed to determine this relationship. Contrastingly, there are positive correlations between thighs asymmetry and high intensity actions (HSR, distance covered between 18 and 21 km/h, > 24 km/h, distance accelerating and Vmax), so accelerating might influence these muscles thermal asymmetries, increasing it, but not as much as in ankles or hamstring whose asymmetry lead players to do not being able to complete the actions. During high-intensity actions in elite football players, various factors can contribute to thermal asymmetries in specific muscles. Firstly, some muscles may be recruited more extensively than others to generate the required force and power, leading to localized heat production and temperature changes in those muscles. Additionally, blood flow distribution may vary during high-intensity activities, with muscles experiencing higher demands receiving increased blood flow to support energy supply and waste removal, leading to temperature variations. Moreover, the increased energy expenditure during intense movements can also contribute to temperature changes in active muscles. Biomechanics and movement patterns play a role as well, as different actions may impose varying stress and strain on muscles, influencing their thermal response. Furthermore, fatigued muscles may exhibit altered thermoregulatory responses, contributing to temperature differences.

For the GPS ratio variables, there are negative correlations statistically significant between thighs and acute-chronic workloads (HSR Coupled, distance covered > 24 km/h coupled, HMLD coupled, and distance accelerating Coupled). On the other hand, there are positive correlations between hamstrings and acute-chronic workloads (Distance accelerating uncoupled, distance > 24 km/h uncoupled, distance > 24 km/h uncoupled). These results may indicate that higher workloads generate hamstring asymmetries, increasing injury risk^12^, while does not seem to have as much influence in thighs.

Finally, for Wellness results, there are negative correlations between Outer Upper Thigh asymmetry and rest quality, Adductor asymmetry and resting time, and Knee asymmetry with resting time. So the lack of rest or bad quality of it, do not allow players to recover well, and can lead to asymmetries of some muscles, increasing injury risk^12^. In addition, there are positives correlation between the Outer Front Thigh asymmetry and muscular soreness, Adductor asymmetry with stress and muscular soreness, and between knee asymmetry and fatigue. So, the previously sleep disturbances commented can also induce more fatigue and muscular soreness, which increase injury and underperformance risk^46^ and more stress, that also intensify injury^47^ and miss performance risk^48^. On the other hand, there is a negative correlation statistically significant between a global value of the body asymmetry with stress, this result go against priory commented results, as players with more global asymmetry should have more stress. This situation requires further research, but perhaps higher stress values influence body temperature and performance but do not generate thermal asymmetries.

For mean temperatures, there are negative correlations where higher acute-chronic workloads appear with lower temperatures. So perhaps higher training loads generate more central fatigue, and it influences the thermoregulation, producing lower temperatures. While the only mean temperature related with external load variables, is from the ankle which correlated with distance covered > 24 km/h, V_max_, and ACC_max_, maybe because sprinting and accelerating generates increased blood flow in the ankle, and that increment temperature, other option is that as sprint actions precede goals^35^ players who performed these actions kicked more times the ball, and impacting the ball increased ankle temperature as this muscle receive much energy and is very important in this action. However, ankle is a very controverted ROI to analyze, and segmentation can influence these results.

Finally, for the relationships between mean skin temperature and wellness, higher mean skin temperatures of the thighs and adductors are related with less muscle soreness and stress. On the other hand, higher mean skin temperatures of the inner back leg are related with less rest, higher knee temperature with more fatigue and worse rest and higher ankle temperature with more stress. There results may indicate that relationship between temperature and wellness in different muscle groups has different implications due to the varying roles and functions of these muscles. For example, higher skin temperatures in the thigh region may promote relaxation and reduce muscle soreness, while higher temperatures in the knee or ankle may increase fatigue or stress in these joints due to their weight-bearing and repetitive motions during activity. This highlights the complex interplay between temperature, muscle function, and overall wellness and the need for further research to fully understand these relationships.

The results of this study should be interpreted with caution as certain limitations need to be considered. First, the asymmetric cut-off was set low, a higher asymmetry cutoff may indicate stronger correlations and significant differences. Second, the sample was so small that future studies should look for exhaustive evidence to confirm that these results hold across different genders, ages, and athletic levels. Also, there are some not accounted variables that can influence results; (1) Variations in weather conditions, might impact the thermal response of players even though the room is always at the same temperature (2) Each player may have unique physiological characteristics and thermoregulatory responses that could influence skin temperature readings (3) Players' stress levels, anxiety, and motivation might impact their physiological responses, including skin temperature. High-stress situations or psychological fatigue could potentially influence the thermal patterns observed. (4) Variations in dietary intake and nutrition among players could impact metabolism and, consequently, skin temperature responses.

In conclusion, High intensity actions contribute to generate more asymmetries in the thighs, so elite coaches should consider decreasing intensity when players show thermal asymmetries on these ROIs. Adductor asymmetries are associated with worse recovery and soreness values, and higher asymmetries in hamstrings difficult players to complete all the high intensity actions necessary to compete at high level. This highlights the importance of controlling muscle asymmetry for optimal wellness and performance. Correlations between muscle asymmetry and wellness have also been observed, suggesting that higher asymmetries produce worse well-being, reinforcing the need for coaches to closely monitor these metrics. Finally, it is also important to consider the mean temperature as it may impact muscle performance and recovery, and thus, should be taken into account by coaches when making decisions about player management.

From a practical point of view, coaches can design training programs based on players' thermal responses and well-being status to optimize performance and reduce injury risk in elite football players. Also, they can integrate regular well-being questionnaires and thermography assessments as they provides valuable insights into their physical and mental readiness, enabling coaches to adjust training loads and implement proper recovery strategies. Finally, considering the influence of temperature in players, future studies could investigate personalized recovery strategies, such as cold-water immersion and massage, based on individual thermal responses.

### Supplementary Information

Supplementary Information.

## Data Availability

All the data of the study can be found in the Supplementary File “SF1. Data of the Study”.
